# Pharmacogenomic Studies of Antiviral Drug Favipiravir

**DOI:** 10.3390/pharmaceutics16040503

**Published:** 2024-04-07

**Authors:** Victoria V. Shumyantseva, Tatiana V. Bulko, Alexey A. Chistov, Ekaterina F. Kolesanova, Lyubov E. Agafonova

**Affiliations:** 1 Institute of Biomedical Chemistry, Pogodinskaya Street, 10, Build 8, Moscow 119121, Russia; tanyabulko@mail.ru (T.V.B.); dobr14@yandex.ru (A.A.C.); ekolesanova@yandex.ru (E.F.K.); agafonovaluba@mail.ru (L.E.A.); 2 Department of Biochemistry, Pirogov Russian National Research Medical University, Ostrovitianov Street, 1, Moscow 117997, Russia

**Keywords:** favipiravir, electrochemistry, modified electrodes, DNA, drug/DNA interaction

## Abstract

In this work, we conducted a study of the interaction between DNA and favipiravir (FAV). This chemotherapeutic compound is an antiviral drug for the treatment of COVID-19 and other infections caused by RNA viruses. This paper examines the electroanalytical characteristics of FAV. The determined concentrations correspond to therapeutically significant ones in the range of 50–500 µM (R^2^ = 0.943). We have shown that FAV can be electro-oxidized around the potential of +0.96 V ÷ +0.98 V (vs. Ag/AgCl). A mechanism for electrochemical oxidation of FAV was proposed. The effect of the drug on DNA was recorded as changes in the intensity of electrochemical oxidation of heterocyclic nucleobases (guanine, adenine and thymine) using screen-printed graphite electrodes modified with single-walled carbon nanotubes and titanium oxide nanoparticles. In this work, the binding constants (Kb) of FAV/dsDNA complexes for guanine, adenine and thymine were calculated. The values of the DNA-mediated electrochemical decline coefficient were calculated as the ratio of the intensity of signals for the electrochemical oxidation of guanine, adenine and thymine in the presence of FAV to the intensity of signals for the electro-oxidation of these bases without drug (S, %). Based on the analysis of electrochemical parameters, values of binding constants and spectral data, intercalation was proposed as the principal mechanism of the antiviral drug FAV interaction with DNA. The interaction with calf thymus DNA also confirmed the intercalation mechanism. However, an additional mode of interaction, such as a damage effect together with electrostatic interactions, was revealed in a prolonged exposure of DNA to FAV.

## 1. Introduction

**Scheme 1:**
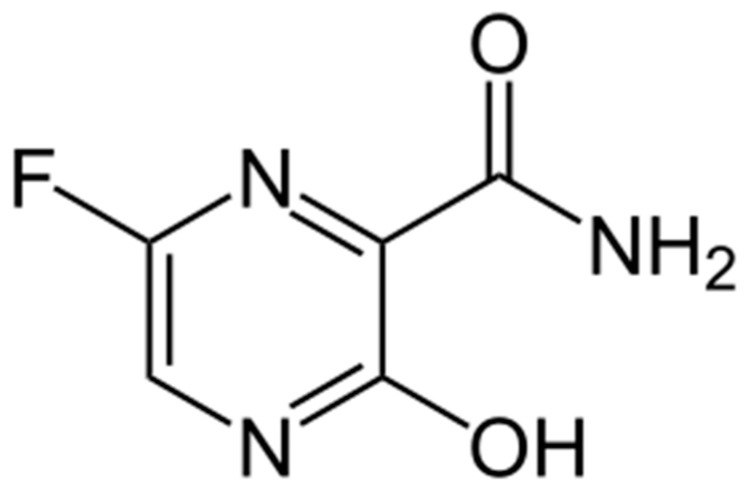
Chemical structure of favipiravir (6-fluoro-3-hydroxypyrazine-2-carboxamide).

Favipiravir’s (6-fluoro-3-hydroxy-1,4-pyrazine-2-carboxamide, Scheme 1) (FAV) is rather broad antiviral activity has led to great interest being shown in it by the research community [1]. This synthetic analog of natural nucleobases shows efficacy against various RNA-containing viruses: orthomyxoviruses (human influenza type A, B and C viruses) [2,3,4,5,6], flaviviruses (Zika, denge and West-Nile fever viruses) [7,8,9], arboviruses (Rift Valley, Crimean-Congo fever viruses) [10,11], arenaviruses [12], paramyxo-, alpha-, bunya-, and noroviruses [13,14]. FAV was found relatively efficient against such dangerous pathogens as Ebola fever [15] and rabies viruses [16].

FAV was shown to effectively inhibit the reproduction of human influenza viruses, including those resistant to neuraminidase and M2 protein inhibitors, as well as potentially dangerous swine and avian influenza viruses [4,5,6,17,18]. A number of clinical trials demonstrated FAV efficacy in treating infections caused by coronaviruses SARS-CoV, MERS and SARS-CoV2 [19,20,21,22]. However, in the course of clinical studies and observations, the side effects of FAV were revealed (nausea and vomiting, hyperuricemia, increased hepatic transaminases, neutro- and leukopenia, in some cases—cardiotoxicity and a number of others) [6,19,20,21,22,23,24]. The results of FAV preclinical studies on small laboratory animals indicated its teratogenicity and embryotoxicity [6]. This imposes restrictions on the use of FAV medications in a number of patients. The side effects are mainly associated with the effect of this compound on the metabolism of nucleobases and potential mutagenicity by interacting with DNA [24].

FAV is a prodrug. It turns into the active compound 5’-ribosyl triphosphate FAV in human body cells [3,5,25,26,27]. This nucleotide analog can be recognized as a substrate by RNA-dependent RNA-polymerases from various viruses and can be incorporated in viral RNA as an analog of 5′-guanilic acid (G) or, less likely, of 5′-adenylic acid (A), thus causing lethal mutations in the viral genome, behaving as a purine analog [28,29]. However, FAV also can form FAV:G and FAV:A pairs, and, in this case, FAV would behave as a pyrimidine analog [29]. This process is considered a principal mechanism of FAV activity against various viruses [28,29,30,31]. During influenza virus replication, FAV can also act as an inhibitor of viral RNA polymerase, causing the formation of truncated RNA chains [25,32]. DNA-dependent RNA polymerases do not recognize FAV ribosyl triphosphate as a substrate and do not incorporate it in transcribed RNA, thus establishing the base for selective effects of FAV on RNA-containing viruses [3].

FAV phosphoribosylation to ribosyl monophosphate nucleotide is catalyzed by hypoxanthine-guanine phosphoribosyl transferase [25,26,27]; the enzymes that are employed in the further phosphorylation up to ribosyl triphosphate FAV are unknown [27]. FAV conversion to the corresponding nucleotide proceeds much slower than in the case of natural nucleotides: monophosphoryl-ribosylation is 4700 times slower on average [26]. In addition, the rates of these conversions depend on cell types [33]. Hence, a substantial part of FAV that penetrates the cells may remain as the nucleobase, and this prodrug form can cause a part of its side effects. Therefore, it was of interest to study the possibility of interaction of FAV with cellular components at the molecular level. Taking into account base-pairing properties of FAV [29], its interaction with DNA by an intercalation between nucleobase residues in the double helix seems possible.

DNA plays an important role in a variety of biological processes such as gene transcription, mutagenesis and carcinogenesis. The interaction of a drug with DNA can help to understand the mechanisms and patterns of pharmacological chemicals actions and to screen DNA-targeting drugs.

Electroanalytical techniques provide useful insights into the mechanisms of the interactions of DNA with medications [34,35,36,37,38,39]. Electroanalysis has a triple function, registering the drug itself, DNA, and the DNA/drug complex. Scrutiny of the electrochemical signals of DNA or DNA/chemotherapeutic agent complex before and after biding can register the interaction and clarify the binding mechanism.

The goal of this research was to assess the impact of FAV as a remedy with a broad spectrum of antiviral action on the DNA molecule via the registration of electro-chemical oxidation signals of heterocyclic bases guanine, adenine, and thymine. The novelty of our research lies in the first investigation of FAV with dsDNA and ctDNA from a pharmacogenomics viewpoint.

## 2. Materials and Methods

### 2.1. Apparatus

In our experiments, we used two types of potentiostat/galvanostat, such as PGSTAT 12 Autolab (Metrohm Autolab, Utrecht, The Netherlands) with the GPES software (version 4.9.7) and a PalmSens potentiostat (PalmSens BV, Houten, The Netherlands) with PSTrace software (version 5.8). Electrochemical measurements were conducted at 20.0 °C. All experiments were performed in a supporting electrolyte—0.1 M potassium phosphate buffer with 50 mM NaCl (PBS, pH 7.4). For the registration of direct analysis of electrochemical dsDNA oxidation, the differential pulse voltammetry (DPV) method was used. The following optimized DPV parameters were used: potential range of 0.2–1.2 V, pulse amplitude of 0.025 V, potential step of 0.005 V, pulse duration of 50 ms, and modulation amplitude of 0.05 V. The square wave voltammetry (SWV) settings were as follows: potential range of 0–1.2 V, pulse amplitude of 0.005 V, potential step of 0.005 V, and frequency 10 Hz. Triplicated measurements were applied for electrochemical estimation of various DNA concentrations. These values were within 10–12% (the standard deviation, SD =10–12%), which satisfies the repeatability of the electrochemical installation when studying biological objects.

The UV–vis absorption spectra were registered with Cary 100 Scan spectrophotometer UV–Vis (Agilent Technologies, Inc., Santa Clara, CA, USA) with the software Cary WinUV version 3.00(182). The absorption spectra for FAV, DNA and the FAV/DNA complex were recorded in the range of 200–500 nm.

Commercially available screen-printed electrodes (SPEs) were obtained from ColorElectronics, Moscow, Russia (http://www.colorel.ru (accessed on 1 February 2024)). The SPEs contained graphite working electrodes (geometric area 0.0314 cm^2^), auxiliary electrodes, and silver/silver chloride reference electrode (Ag/AgCl). All potentials were referred to the Ag/AgCl reference electrode.

### 2.2. Chemicals

“Medsintez” Ltd. (Moscow, Russia) kindly provided Favipiravir drug substance. Single-walled carbon nanotubes (SWCNT, diameter 1.6 ± 0.4 nm, length > 5 µM, surface area 1000 m^2^/g) TUBALL™ BATT H_2_O, with water dispersion of 0.4%, stabilized by carboxymethylcellulose, were obtained from OCSIAL Ltd. (https://ocsial.com, (accessed on 1 February 2024)). Potassium phosphate monobasic (≥99%), and potassium phosphate dibasic trihydrate (≥99%) were purchased from Sigma-Aldrich. Sodium chloride (99.5%) was purchased from Acros Organics (Carlsbad, CA, USA). Double-stranded fish sperm DNA (dsDNA) as lyophilized powder was bought from Sigma-Aldrich (D 3159, Tokyo, Japan). Calf thymus DNA was obtained from Serva (Heidelberg, Germany). All other chemicals were of analytical grade and used without further purification. All aqueous solutions were prepared using Milli-Q water (18.2 MΩcm) purified with a Milli-Q water purification system by Millipore. The quality and purity of the DNA stock solution was checked by taking the absorbance ratio of A_260_/A_280_, which was found to be in the range of 1.8–1.9, indicating there is no contamination of protein in ds-DNA solution [39]. The stock solution of dsDNA (3 mg/mL) was prepared in 100 mM potassium phosphate buffer with 50 mM NaCl (PBS, pH 7.4) [40,41].

### 2.3. Preparation of Modified Electrode

To modify the surface of working electrodes with single-walled carbon nanotubes, we applied 2 μL of dispersion of SWCNT by drop casting. The commercial dispersion of SWCNT TUBALL™ BATT H_2_O was preliminarily diluted 5 times in distilled water (named SPE/CNT; 0.75 ± 0.05 mg/mL). The concentration of SWCNT was chosen empirically as the best signal-to-noise ratio.

For surface modification with titanium oxide, the TiO_2_ suspension (1 mg in 0.5 mL of H_2_O:C_2_H_5_OH mixture, 1:1 *v*/*v* [42,43]) was sonicated for 1 h and a 2 μL aliquot of the suspension was pre-mixed with 2 μL of dispersion of SWCNT. SPE/CNT/TiO_2_ was prepared by drop casting of 4 μL mixture of CNT and TiO_2_ suspensions.

Electrodes SPE/CNT and SPE/CNT/TiO_2_ stayed at room temperature until completely dried. Modified SPEs were used for a single measurement in order to avoid the blockage of the electrode surface by oxidation products of DNA or FAV.

For noncovalent immobilization of dsDNA, 60 µL of the dsDNA solution (1.5 mg/mL) prepared in PBS, with pH 7.4, was dropped onto the surface of the modified electrode and incubated for 15 min before measurements. For the investigation of DNA/drug interaction, a complex of DNA with FAV was formed at the constant concentration of DNA 1.5 mg/mL and specified concentrations of FAV, and was incubated 40 min before adsorption onto the electrode surface. DPV measurements were performed after 60 s deposition time.

A horizontal measurement regimen was used for all electrochemical experiments. A 60 μL drop of PBS was placed onto the SPEs to cover the surface of all three electrodes. Experiments were performed under aerobic conditions at room temperature (25 ± 3 °C). To assess the reproducibility of the results for each concentration, at least 3 electrodes were used and the standard deviation was calculated.

The calibration curve as kSD/b (k = 3 for the limit of detection, LOD, b = slope) was employed for the calculation of LOD (SD =standard deviation of the intercept) [44,45].

Linear plots for 1/(I_0_ − I) depending on 1/[FAV] were used for the calculation of the binding constant Kb [40,41] in accordance with the following equation: Kb ≈ (I_0_ − I)/(I_0_ × ([FAV] − (I_0_ − I)).

## 3. Results and Discussion

### 3.1. Electrochemical Profiling of Favipiravir on SPE/CNT and SPE/CNT/TiO_2_

The rising interest in FAV is ascribed to its efficient suppression of dangerous and even lethal infections caused by numerous viruses, such as hemorrhagic fevers, COVID-19, SARS, MERS, rabies, etc. [8,9,10,11,12,13,14,15,16,17,18,19,20,21,22,23,24,25,26,27,28,29,30,31,32,33], and by its possible efficacy against viruses, which can be transferred to humans from animals and have a high epidemic potential [1,4,5,6,17,18]. Several drug preparations of FAV have been already developed and registered for clinical use (T-705, Avigan, Favilavir, Avifavir, Areplivir, Coronavir) [5,20,21,22] and many derivatives and analogs are under development (reviewed in [46]). Clinical usage stimulates FAV research in the field of its side effects and the mechanisms involved therein, and the elaboration of methods of drug control and determination in organs, tissues and the environment.

For quantitative determination of FAV in biological fluids and in pharmaceutical formulations, environmental samples have been collected and several methods have been described, such as UV–Vis spectrophotometry, spectrofluorimetric method, high-performance liquid chromatography (HPLC), and liquid chromatography–tandem mass spectrometry (LC–MS/MS) [47,48]. Electrochemical methods offer great advantages, such as high sensitivity, elaboration of the instrumental equipment with friendly software, quick response rate, usage of low-toxicity reagents, such as aqueous electrolyte buffer solutions, and miniaturization mode for the analysis in a “point-of-care” regimen [49,50].

In our experiments, we used disposable screen-printed electrodes as commercially available transducers, with a relatively low cost, to explore in clinical laboratories their suitability for modifications with different types of nanomaterials [51,52]. Electrochemical profiling of FAV on the screen-printed electrodes modified by single-walled carbon nanotubes (SPE/CNT) and carbon nanotubes with nanosized TiO_2_ was studied. Carbon nanomaterials, such as carbon nanotubes and carbon nanotubes with nanosized titanium (IV) oxide TiO_2,_ significantly improve the sensitivity of electrodes [53,54,55,56]. TiO_2_ in various forms (nanoparticles, nanotubes, nanoneedles) has properties that make it attractive for the modification of electrodes, such as biocompatibility, specific binding to biomolecules owing to amphoteric chemical nature, optical transparency, large specific surface area for high biomolecule content, and a stimulating effect for electron transfer between the electrode and molecule under investigation [56]. Previously, we employed/applied CNT + titanium (IV) oxide nanoparticles for protein and amino acid immobilization on electrode surface for the fabrication of electrochemical biosensors based on the electrochemical oxidation of amino acids, such as tyrosine, tryptophan, histidine, methionine, and cysteine [42,43]. In our study, we used this nanomaterial for the electrochemical analysis of FAV. Before the measurements, we used adsorptive accumulation of favipiravir on the electrode surface within 40 min. The electrochemical behavior of FAV was studied in physiologically relevant PBS, with pH 7.4.

Electrochemical analyses were performed using cyclic voltammetry (CV) and the differential pulse voltammetry (DPV) technique. Disposable carbon SPEs modified with CNT (SPE/CNT) and CNT/TiO_2_ (SPE/CNT/TiO_2_) were used for CV and DPV, respectively. CVs for the 500 µM FAV in the potential range of 0.6 V÷ +1.2 V were analyzed in electrolyte PBS (pH 7.4).

**Figure 1:**
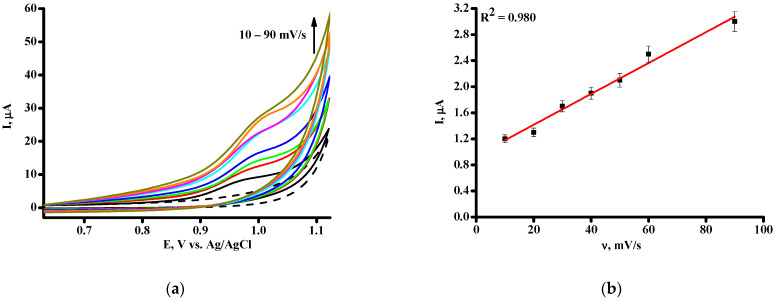

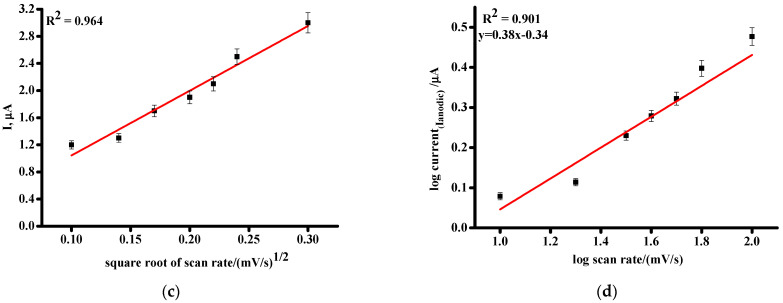
(**a**) CV of 500 µM FAV on SPE/CNT at different scan rates from 10 to 90 mV/s, (-), 10 mV/s); (-), 20 mV/s); (-) 40 mV/s; (-), 50 mV/s); (-) 60 mV/s); (-), 70 mV/s); (-), 80 mV/s); (-), 90 mV/s; (**b**) the linear plot of the oxidation peak current of FAV vs. the scan rate; (**c**) the linear plot of the oxidation peak current of FAV vs. the square root of the scan rate; (**d**) Log(Ip) dependence on Log (ν).

During the anodic scan from 0.6 to +1.2 V, one broad oxidation peak was received at around +0.98 V and +0.96 V for SPE/CNT and SPE/CNT/TiO_2_, respectively, while no reduction peak was observed in the reverse cathodic scan (Figure 1a). The peak at E = +0.98 V ÷ +0.96 V of FAV could reflect the oxidation of the hydroxyl group to the keto group, as was shown earlier [48,49].

The oxidation peak potential (E_pa_) of FAV recorded by cyclic voltammetry shifted towards the positive direction, with the increase in the scan rate as an additional characteristic of an irreversible electrode reaction (Figure 1a) [57,58,59,60,61]. We registered a linear relationship between the oxidation peak current and the scan rate ν, and between the oxidation peak current and square root of scan rate ν^1/2^ for SPE/CNT (Figure 1b,c) and for SPE/CNT/TiO_2_.These experimental dependences indicated that the FAV electrochemical oxidation process revealed a mixed mechanism, controlled by both adsorption and diffusion [57,58,59,60,61]. The same properties of the FAV electrochemical oxidation process were also demonstrated while using boron-doped diamond electrodes and carbon electrodes [47,48,62]. The slope of the log Ipa against log *v* is 0.38 (Figure 1d); the equation’s slope confirmed that the FAV electrochemical oxidation process revealed a mixed mechanism controlled by a mixed diffusion-controlled electrochemical reaction [57,58,59,60,61].

The irreversibility of the oxidation process was also confirmed by means of linear dependence of plot of (E) on logarithmic (ν) in accordance with Laviron’s theory (Figure S1) [47,48,60,61,62,63,64,65,66].

**Figure 2:**
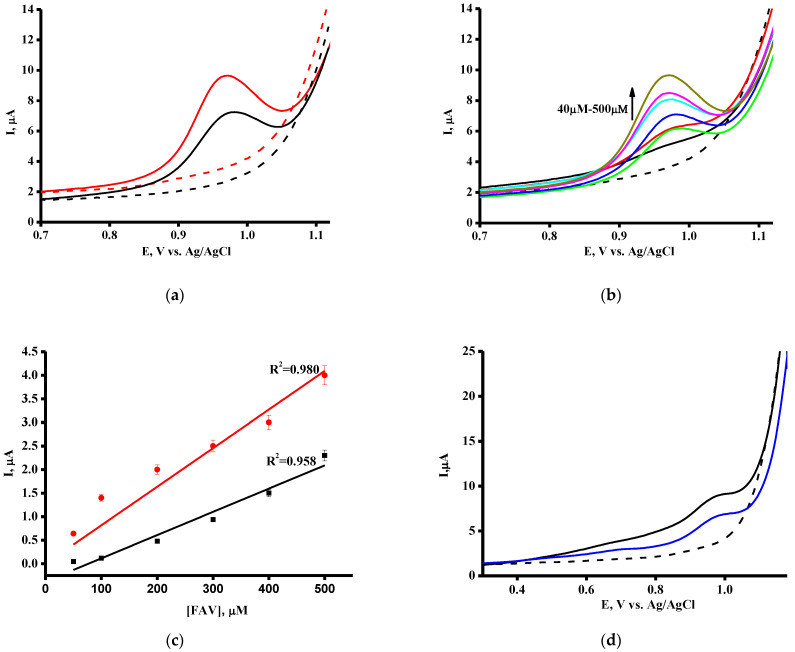
(**a**) Comparative DPV curves of 500 µM FAV on SPE/CNT (black) and SPE/CNT/TiO_2_ (red), (---) and (---) curves corresponded to blank electrodes; (**b**) DP voltammograms recorded in 0.1 M potassium phosphate electrolyte buffer, pH 7.4 (PBS) with increasing FAV concentration in the range of 50–500 µM (-), 40 µM; (-) 100 µM; (-), 150 µM; (-), 200 µM; (-) 300 µM; (-), 400 µM; (-), 500 µM. Dashed black line represents DPV of SPE/CNT/TiO_2_ in PBS. The standard deviation of the DPV response on the SPE/CNT/TiO_2_ for three experiments was ± 5%; (**c**) DPV peak current versus the concentration of FAV for SPE/CNT (black) and SPE/CNT/TiO_2_ (red); (**d**) DPV of first (-) and second (-) scan of 50 µM SPE/CNT/TiO_2_.

Differential pulse voltammetry (DPV), one of the most sensitive and contemporary electrochemical techniques, possesses high analytical sensitivity, which permits us to register heterocyclic nucleic bases separately for the detailed analysis of intricate drug/DNA interaction mechanism. The DPV of FAV in the potential range of +0.6 V÷ +1.2 V was investigated in electrolyte PBS. The DPV of FAV revealed a single anodic peak at E = +0.972 ± 0.003 V and E = +0.967 ± 0.003 V (Figure 2a) for SPE/CNT and for SPE/CNT/TiO_2_, respectively.

Favipiravir dosing regimens differ for different viral infections. To achieve drug efficacy, medical doses are high and correspond to the micromolar region [46,62]. In our experiments, we used a pharmacologically relevant FAV concentration range from 50 to 500 µM [5,19,20,21,22]. For the range of 50–500 µM, the regression equations were I = (0.0049 ± 0.0005) [FAV] − 0.38 ± 0.10 and I = (0.0082 ± 0.0005) [FAV] + 0.52 ± 0.10 for SPE/CNT or SPE/CNT/TiO_2_, respectively (Figure 2b,c). The described method was validated for parameters such as linearity and limits of detection (LOD). Electroanalytical parameters of quantitative FAV analysis with SPE/CNT and SPE/CNT/TiO_2_ are presented in Table 1. The sensitivity of SPE/CNT/TiO_2_ was approximately twice as high in comparison with SPE/CNT.

The calculation using Equation (1) and DPV parameters for FAV on SPE/CNT or SPE/CNT/TiO_2_ and [67,68] showed that one electron is involved in the oxidation process of this drug [48,62,63,69]:

W _1/2_ = 3.52 RT/nF (1)

where we used common abbreviations for parameters, as W _1/2_ is the DPV half peak width, R is gas constant, 8.3145 J K^−1^mol^−1^, T is temperature, in Kelvin, F is Faraday constant 96,485 C mol^−1^, and n is the number of electrons involved in the oxidation reaction.

The irreversibility of FAV electrochemical oxidation was also confirmed via comparing the first and second scan of DPV, which demonstrated the decline in oxidation current corresponding to the second scan (Figure 2d). On the other hand, the decrease in oxidation peak current might be explained by partial fouling and inactivation of the available electrode surface area [62,63,64,65,66,67].

Earlier, it was shown that FAV could be analyzed electrochemically using different type of electrodes and modifications [47,48,49,50,62,63,69]. FAV can be electro-oxidized using a bimetallic nanocomposite based on gold/silver core–shell nanoparticles with conductive polymer poly (3,4-ethylenedioxythiophene) polystyrene sulfonate and functionalized multi-carbon nanotubes on a glassy carbon electrode. DPV technique and the working potential of 1.25 V with two linear ranges from 0.005 to 0.009 and 0.009 to 1.95 μM with a limit of detection of 0.46 nM (S/N = 3) were described [47]. Boron-doped diamond electrodes [48] and screen-printed electrodes modified by means of MnO_2_/graphene derivatives [45] were used for the electroanalytical sensing of FAV at the potential of 1.23 V. A comparison of the electroanalytical methods for FAV analysis is given in Table S1.

### 3.2. Investigation of the Interaction between Favipiravir and dsDNA

Binding models for the interaction between dsDNA and drugs classify them as electrostatic interactions, including binding in minor or major grooves and intercalative binding [36,37,38,68,70].

**Figure 3:**
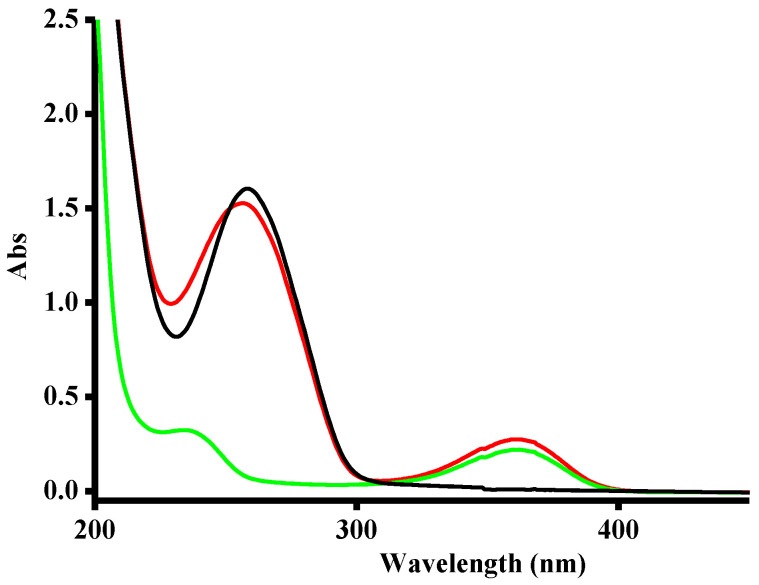
Absorption spectra in presence of 0.075 mg/mL dsDNA (black), 25 μM FAV (green), and FAV/dsDNA complex (red) in PBS.

Spectroscopic methods are the traditional technique for the investigation of drug/DNA interactions from the pharmacogenomics viewpoint. We analyzed the UV-vis absorption spectra of FAV, dsDNA and the complex of FAV/DNA (Figure 3). FAV shows two absorption peaks at 234 nm and 362 nm. When complex FAV/DNA was formed, a hypochromic effect (i.e., a decrease in absorbance) at 257 nm was recorded together with a slight hypsochromic blue shift. As can be seen from Figure 3, FAV, FAV/DNA complex and DNA have absorption spectra with close maximum wavelengths. Therefore, the explanation of the mechanism of drug/DNA interaction is ambiguous. However, based on the hypochromic effect, it is possible to propose that FAV interacts with DNA in accordance with the intercalative mechanism [69].

**Figure 4:**
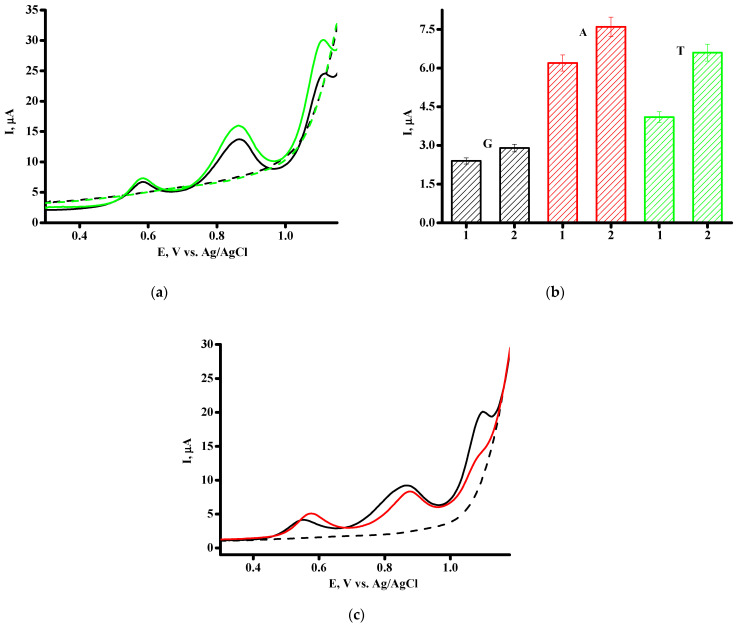
(**a**) DPV of dsDNA (1.5 mg/mL) on SPE/CNT (black line) and SPE/CNT/TiO_2_ (green line), blank SPE/CNT (---) and SPE/CNT/TiO_2_ (---); (**b**) histogram of oxidation peak current for dsDNA (1.5 mg/mL) for SPE/CNT (1) and SPE/CNT/TiO_2_ (2); (**c**) DPV of first (black line) and second scan (red line) of dsDNA (1.5 mg/mL) on SPE/CNT/TiO_2_.

The electrochemical technique was used for the investigation of the FAV/dsDNA interaction mechanism as a sensitive and robust technology. Earlier, we showed that electrochemical oxidation of dsDNA was successfully achieved on SPE/CNT after dsDNA immobilization by physical entrapment/absorption on the surface of the modified electrodes. Modification of SPEswith CNT stabilized with carboxymethylcellulose (SPE/CNT) allowed the registering of electrochemical oxidation of guanine, adenine and thymine in the immobilized dsDNA [40,41,71]. Adding nanosized titanium (IV) oxide TiO_2_ improved the sensitivity of electrodes for the registration of dsDNA (Figure 4a,b), as in the case of protein registration [43]. Based on this data, we used SPE/CNT/TiO_2_ for the investigation of interaction between FAV and dsDNA. We carried out electrochemical measurements with the DPV method. DPV was chosen as a sensitive and informative technique for drug/DNA assay. The main advantage of DPV is also a significant decline in the contribution of capacitive current in comparison with faradaic current.

DPV of the first (black line) and second (red line) scan of dsDNA (1.5 mg/mL) on SPE/CNT/TiO_2_ demonstrated the irreversible nature of the electrochemical process and fouling of the electrode by means of DNA or FAV oxidation products (Figure 4c) [71,72]. Based on this observation, we used SPEs only for single measurements. This regimen is typical for the irreversible electrochemical process [40,41,73].

DNA carries genetic information required for the synthesis of proteins. Apart from this role of DNA, different types of DNA or RNA molecules may be present in normal and pathological cells and fluids in human body, such as circulating tumor DNA (ctDNA), microRNA, therapeutic nucleic acids and cell free DNA (cfDNA). These types of DNA or RNA possess diagnostic relevance as specific markers of diseases and play a role in organism defense at the transcription/translation regulation level [72,74,75,76,77]. The detection of DNA/drug complex formation and study of the mode of interactions are the main points of pharmacogenomics [36,78,79,80,81,82,83].

**Figure 5:**
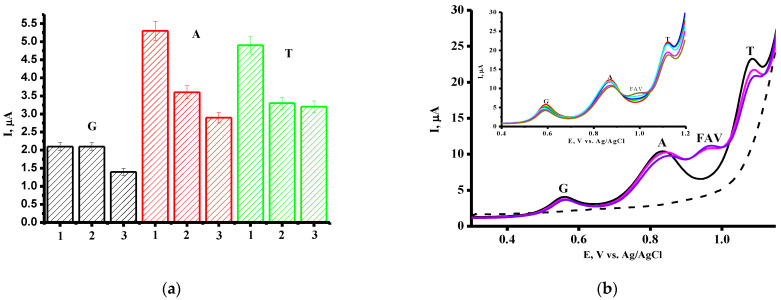
(**a**) The average guanine, adenine and thymine signals after interaction with 500 µM FAV at 0 min (1), 5 min (2) and 40 min (3); (**b**) DPV of dsDNA (1.5 mg/mL) on SPE/CNT/TiO_2_ (black line), blank SPE/CNT/TiO_2_ (---) and FAV/dsDNA complex with 400 µM FAV concentration (-), 500 µM FAV concentration (-). Inset: DP voltammograms of dsDNA in the presence of FAV in the concentration range of 50–500 µM (from (-, 50 µM) to (-, 500 µM).

FAV itself demonstrated a DPV oxidation signal at around + 0.96 ÷+1 V, in the region of non-overlapping potentials with nucleobases oxidation potentials. Therefore, the investigation of binding of FAV with dsDNA via electrochemical methods was more effective in comparison with the spectrophotometric technique by means of the observation of changes in peak current intensity of guanine, adenine and thymine of DNA in the presence of FAV (Figure 5a).

Electrochemical analysis of dsDNA based on direct electrochemical oxidation of guanine, adenine and thymine residues [82] was used. This technique was applied for the analysis of the interactions of FAV with DNA. In our designed experimental approach, we used the increased concentration of FAV in the range of 50–500 μM and registered the DPV peak current intensity of G, A and T oxidation of a constant concentration of dsDNA (1.5 mg/mL) immobilized on single-use SPE/CNT/TiO_2_. It is a well-known viewpoint that the positive shift of the oxidation of heterocyclic bases peak potentials is typical for the intercalative hydrophobic mode of drugs during binding to DNA. In contrast, the negative shift is representative of the binding modes such as electrostatic interaction or groove binding [35,36,37,38,80,83]. In order to determine the optimum time for FAV/dsDNA complex formation, we compare the intensities of nucleic bases´ oxidation signals after the interaction of dsDNA with 500 μM in time intervals of 5 and 40 min. The main question of complex formation in a non-covalent system with a reversible equilibrium is to find the balance between complex formation and complex dissociation. We have shown that 40 min interaction time is optimum for an FAV/dsDNA complex (Figure 5a). For this reason, we incubated the complex for 40 min before electrochemical measuring.

The adequacy of the proposed approach was evaluated by experiments in which the dsDNA sensor was incubated in phosphate buffer pH 7.4 without adding FAV. No changes in G, A or T oxidative peak current intensities were observed. In contrast, the interaction of dsDNA with FAV is accompanied by a decrease in the peak current intensities of G, A and T heterocyclic bases (Figure 5b). The influence of FAV is accompanied by a shift of the oxidation potentials of the heterocyclic bases of G, A and T in the anodic direction, registered as 4 ± 2 mV, 10 ± 2 mV and 5 ± 2 mV, respectively. The most pronounced shift was registered for adenine oxidation. Based on this experimental data, it is possible to assume that FAV interacts more intensively with adenine than with guanine or thymine. Positive shifts, observed for registered heterocyclic bases, corresponded to the intercalative mode of drug/DNA interaction mechanisms, as was confirmed during the study of numerous examples [48,49,50,51,52].

Investigation of the mechanism of the FAV inhibition mode revealed that FAV can form FAV:G and FAV:A pairs with pyrimidine-like analog behavior [29]. To estimate the mode of interaction, the binding constants for the complex FAV/dsDNA were calculated based on the values of the oxidation current of nucleobases before and after the interaction of DNA with the drug, in accordance with an earlier-published approach for the determination of this parameter [54,72] (Figure S2). Intercalation-based interactions are characterized by high Kb values, usually 10^4^–10^6^ M^−1^, while lower Kb values imply a rather weaker interaction, such as groove or electrostatic interactions [36,38,78,79,80]. The structure of FAV permits the formation of two hydrogen bonds with adenine residue. The Watson–Crick hydrogen bonding in a canonical G-C pair requires the formation of three bonds; however, FAV cannot constitute exactly three bonds. Based on this speculation, it is possible to explain the most intensive interaction of FAV with the adenine nucleobase [29].

The values of Kb confirmed the intercalative mode of FAV/DNA interaction (Table 2). The changes in Gibbs free energy, ΔG, were found to be negative, and confirmed that FAV/DNA interaction was spontaneous and energetically favorable (Table 2).

**Figure 6:**
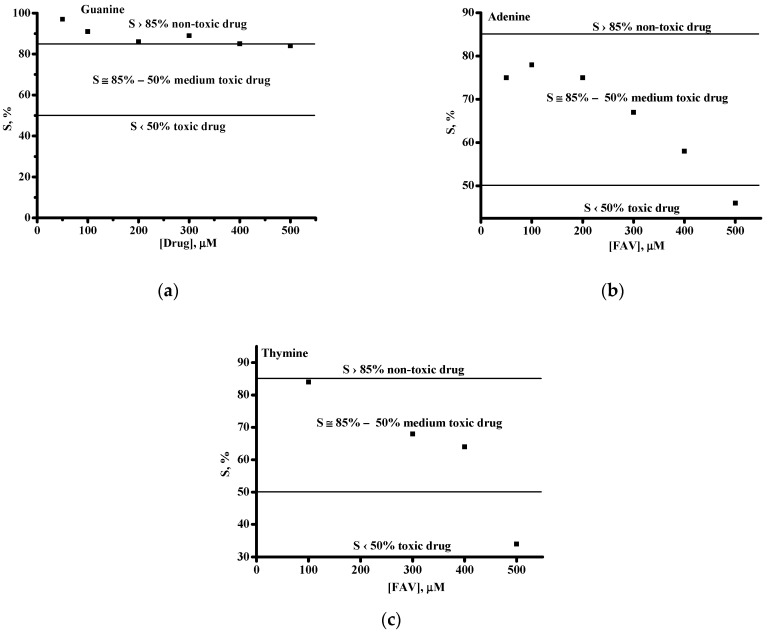
DNA-mediated electrochemical coefficient of toxic effect, S(%), of FAV in the concentration range of 50–500 µM on the signals of electrooxidation of guanine (**a**), adenine (**b**) and thymine (**c**) heterocyclic bases.

The influence of FAV was also represented as the DNA-mediated electrochemical coefficient of toxicity using Equation (2):

S = (Ss/Sb) × 100%(2)

where Sb and Ss are nucleobase oxidation signals before and after interaction of the FAV with dsDNA, respectively [40,41,84]. We used this criterion such that if a drug does not have a toxic effect, S is higher than 85%; it has a moderate toxic effect if the S parameter is between 50 and 85%, and has a toxic effect if S is below 50% [83]. FAV manifested a toxic effect only at a 500 μM concentration (Figure 6a–c).

**Figure 7:**
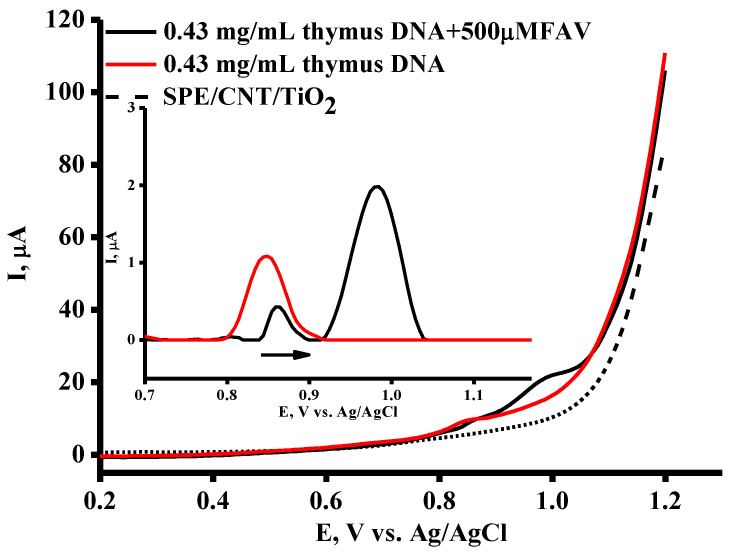
SW voltammograms of 0.43 mg/mL ctDNA (-) and 0.43 mg/mL ctDNA + 500 µM FAV (-); SPE/CNT (---). Inset: the current intensities of the base line corrected SWV peaks for electrochemical oxidation of the ctDNA (-) and for complex ctDNA+500 µM FAV (-).

Double-stranded DNA from calf thymus (ctDNA) was also used as a molecular bio model for the investigation of the interaction with FAV. A total of 2 μL of samples was allowed to rest for 10 min at +37 °C on the surface of the SPE/CNT/TiO_2_ electrodes before measurements. A horizontal measurement regimen in a 60 μL drop of PBS placed onto the SPE to cover the surface of all three electrodes was used for all measurements. As can be seen from Figure 7, the SW voltammogram of ctDNA revealed only one broad peak, centered at a potential of +0.84 ± 0.01 V, and under complex formation, the FAV/ctDNA peak potential shifted by 0.03 V to the more positive direction (0.87 ± 0.01 V), confirming the intercalative mode of interaction with FAV. In comparison with low-molecular-weight dsDNA from salmon sperm, demonstrating three clear peaks, calf thymus DNA exhibited only one peak due to the more compact structure of the DNA molecule [82,83,84]. Investigation of the interaction of the drug with dsDNA permits us to register G, A and T separately and to register the electrochemical response of each base upon drug intake.

**Figure 8:**
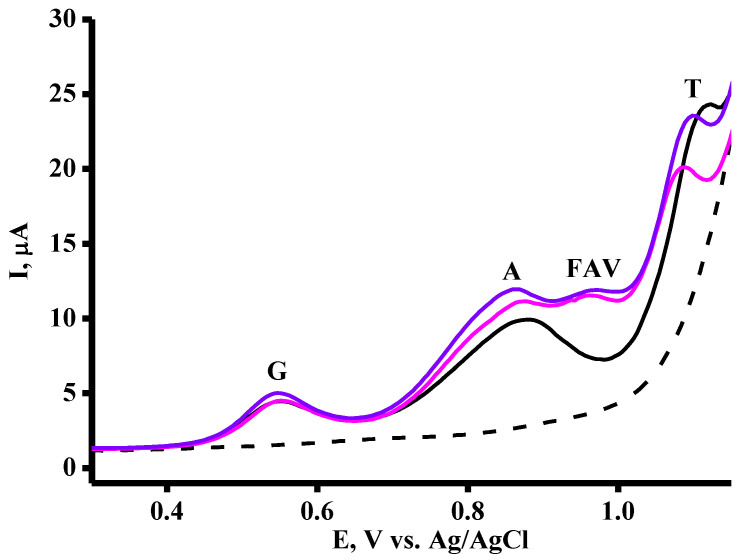
DPV of dsDNA (1.5 mg/mL) on SPE/CNT/TiO_2_ (black line), blank SPE/CNT/TiO_2_ (---) and FAV/dsDNA complex with 400 µM (-) and 500 µM FAV concentrations (-) after 24 h incubation.

Adverse effects of FAV, such as genotoxicity and oxidative stress, were investigated at the molecular level in cell models [23]. When H9c2 cardiomyoblasts or CCD-1079Sk skin fibroblasts were treated with FAV for 24 h, significant DNA damage was registered with Comet assay at a 400 μM FAV concentration, confirming the genotoxic effects of FAV [23]. In our experiments, we incubated dsDNA with FAV (50–500 μM) for 24 h and observed an increase in the oxidative peak current of the heterocyclic bases with negative shifts of oxidation potentials, especially pronounced for A and T residue (registered as 3 ± 1 mV (G), 13 ± 2 mV (A) and 15 ± 2 mV (T) (Figure 8).

The increase in current may reflects the damage to DNA during prolonged incubation with FAV. The negative shift of oxidation potential during 24 h FAV treatment with DNA reflects the transition of the binding mode of DNA/drug interaction from intercalative to electrostatic attraction-assisted groove-binding interactions. Based on the chemical structure of FAV as 6-fluoro-3-hydroxypyrazine-2-carboxamide (Scheme 1), it is possible to assume that this drug not only intercalates into DNA molecule but also forms hydrogen and ionic bonds through NH_2_- and HO- groups as a secondary process of the prolonged incubation of FAV with dsDNA.

## 4. Conclusions

Favipiravir (FAV) is an effective antiviral medication for curing COVID-19 and other infections caused by RNA viruses. However, the interaction of this drug with dsDNA had not been studied earlier. The next focus of our experiments will be the investigation of the pharmacogenomic properties of favipiravir in the process of drug/DNA complex formation. Electrochemistry, as a modern and sensitive platform for the analysis of drug/DNA interactions, possesses remarkable advantages, such as miniaturization, high sensitivity and broad potential window permitting registration of the drug itself and the DNA response as oxidation signals of nucleic bases [35,36,37,38,41,42,81,83,84]. This study utilizes single-use electrodes (SPE/CNT/TiO_2_/DNA) as sensing elements for the investigation of the antiviral drug favipiravir’s interaction with dsDNA. Voltammetric detection of DNA before and after the concentration-dependent drug complex formation permits the calculation of the binding constants Kb of FAV/dsDNA complexes for guanine, adenine and thymine. The qualitative characteristics of FAV/dsDNA interaction, such as the DNA-mediated electrochemical coefficient of toxicity, were also determined. FAV revealed non-toxic or moderate toxic effects in the concentration range of 50–400 µM. Based on the values of the equilibrium constant Kb as 10^4^M^−1^ and shifts of the oxidation potentials of heterocyclic nucleobases to the anodic direction (4÷10 mV), we concluded that FAV interacted with DNA via an intercalative mode. We registered the most pronounced effect for adenine in comparison with guanine and thymine residues. The changes in Gibbs free energy ΔG were calculated as negative values, confirming the spontaneous process of complex formation. Favipiravir’s adverse effect during 24 h incubation with dsDNA was shown. The increase in the oxidation current of DNA could reflect the damage to DNA during the prolonged incubation with FAV.

The novelty of our study is the elucidation of the structural changes in DNA after interaction with the antiviral drug favipiravir using a DNA detection approach based on an electrochemical DNA biosensor. In our investigation, we used a new composite material for electrode modification, carbon nanotubes with titanium oxide nanoparticles, allowing us to improve the sensitivity of the analysis. For registration of binding events as models of pharmacogenomics, we used dsDNA from fish sperm and calf thymus DNA. We discovered that favipiravir showed a mechanism that is more complicated during prolonged incubation with DNA. Monitoring of drug/DNA interactions by means of an electrochemical technique has great promise and may help in the development of new pharmaceuticals.

## Figures and Tables

**Table 1:** Electroanalytical parameters of DPV for FAV determination with SPE/CNT and SPE/CNT/TiO_2_.

Parameters	SPE/CNT	SPE/CNT/TiO_2_
E_ox_, V	0.972 ± 0.003	0.967 ± 0.003
Sensitivity, µA/µM (Slope)	0.0049	0.0082
Linear range, µM	50–500	50–500
LOD, µM	60	37
Equation for linear regression ^1^	Iox = (0.0049 ± 0.0005) [FAV] − 0.38 ± 0.10	Iox = (0.0082 ± 0.0005) [FAV] + 0.52 ± 0.10
Correlation coefficient, R^2^	0.958	0.980

^1^ Iox corresponds to the oxidative currents (peak heights) for FAV.

**Table 2:** Values of FAV/dsDNA binding constants (Kb) and the Gibbs free energy (ΔG).

FAV/dsDNA	K_b_, M^−1^	ΔG = −RTln K_b_, kJ/mol
Based on G oxidation signals	0.24 × 10^4^	−18.96
Based on A oxidation signals	1.03 × 10^4^	−22.51
Based on T oxidation signals	0.20 × 10^4^	−18.52

## Data Availability

The data presented in this study are available in this article (and Supplementary Materials).

## Supplementary Materials

The following supporting information can be downloaded at: https://www.mdpi.com/article/10.3390/pharmaceutics16040503/s1, Figure S1: The relationship between Epa vs. ln ν at the surface of on SPE/CNT; Figure S2: Linear plots for 1/(I_0_ − I) depending on 1/[drug]: (a) FAV/dsDNA (for guanine signals); equation for linear fit: y = 718x + 1.75*10^6^, R^2^ = 0.899, Kb = 0.24*10^4^ M^−1^; (b) FAV/dsDNA (for adenine signals); equation for linear fit: y = 221x + 2.29; R^2^ = 0.917, Kb = 1.03*10^4^ M^−1^; (c) FAV/dsDNA (for thymine signals); equation for linear fit: y = 120.45x + 241154, R^2^ = 0.927, Kb = 0.20*10^4^M^−1^; Table S1: Electrochemical FAV detection based on electro oxidation.
