# Supplementary material for: Pharmacogenomic Studies of Antiviral Drug Favipiravir
Source: Pharmaceutics. 2024 Apr 7;16(4):503. doi: 10.3390/pharmaceutics16040503 (PMC11053860; doi:10.3390/pharmaceutics16040503)
Supplement: Supplementary file 1 [file pharmaceutics-16-00503-s001.zip › pharmaceutics-2916416-supplementary.pdf]

Supplementary Materials

Table S1. Electrochemical FAV detection based on electro oxidation.

**Method**	**Type of electrode (Modification)**	**Linear range, µM**	**Limit of detection, µM**	**Reference**
SWV	BDDE	0.064–130	0.018	[49]
SWV	MnO_2-rGO/SPE	0.01–55	0.009	[50]
DPV	GCE (bimetallic)	0.005–2.0	0.0046	[48]
	gold/silver core–shell nanoparticles (Au@Ag CSNPs)			
DPV	Pencil graphite electrode	5.0–200.0 (1st linear segment)	1.55 (oxidation)	[51]
	PGE	200.0–600.0 (2nd linear segment)		
DPV	Diamond NPs/CPE	0.2-1.0	4.83×10^{-3}	[64]
AdsSWV	GCE	6.4–640	1.7	[65]
Electrochemical impedance spectroscopy	Fe3O4@SiO2–Au/GCE	0.1–104 ng/mL	4.78 pg/mL	[66]

The CV and AdS-SWV techniques	Carbon black (Au@CCB) modified graphite nanopowder flakes paste electrode (GNFPE)	0.03 – 77.3	8.1 nM	[63]
SWV	(Pt@rGO/ GCE	3.16 to 100.0 µM	2.46 µM	[67]
DPV	SPE	50 - 500	37 µM	This work

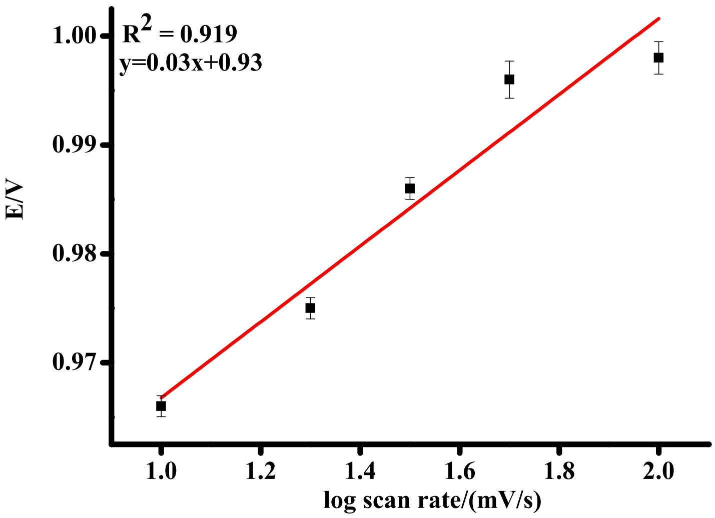

Figure S1. The relationship between Epa vs. ln ν at the surface of on SPE/CNT.

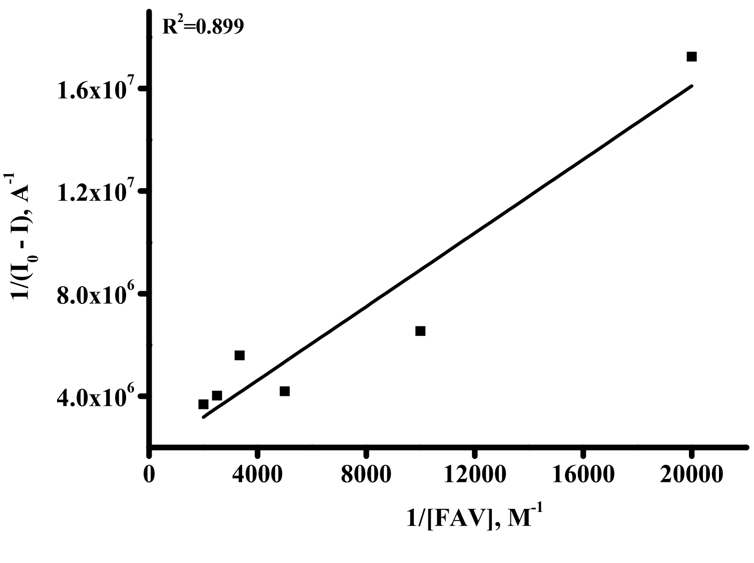
R²=0.899
1.6x10⁷
1.2x10⁷
8.0x10⁶
4.0x10⁶
1/(I₀ - I), A⁻¹
0
4000
8000
12000
16000
20000
1/[FAV], M⁻¹

(**a**)

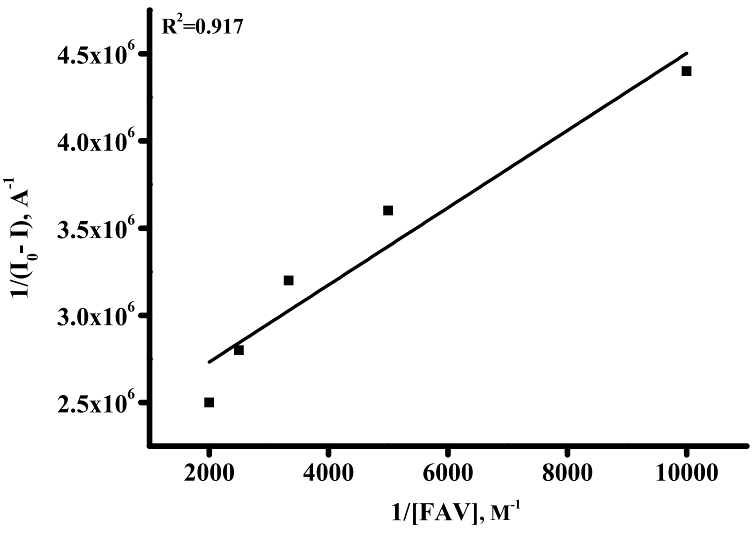
R²=0.917
4.5x10⁶
4.0x10⁶
3.5x10⁶
3.0x10⁶
2.5x10⁶
1/(I₀ - I), A⁻¹
2000
4000
6000
8000
10000
1/[FAV], M⁻¹

(**b**)

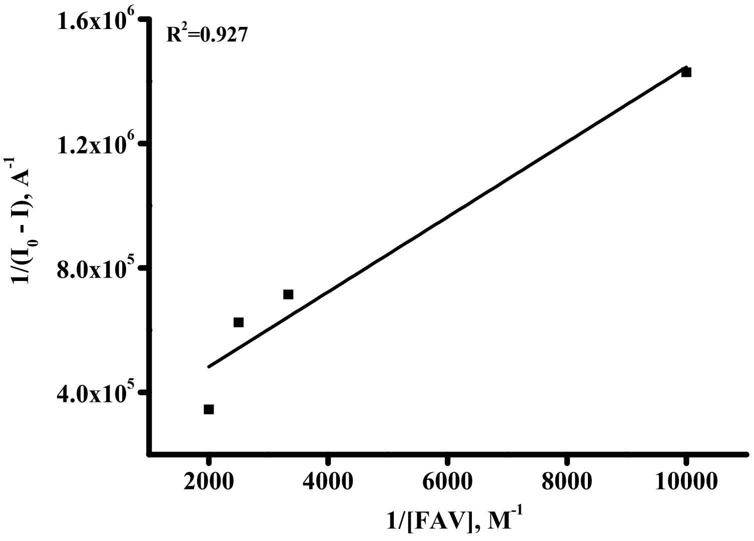

(**c**)

Figure S2. Linear plots for $1/(I_0 - I)$ depending on 1/[drug]: (**a**) FAV/dsDNA (for guanine signals); equation for linear fit: $y=718x+1.75\times10^6$, $R^2=0.899$, $Kb=0.24\times10^4\ M^{-1}$;

(**b**) FAV/dsDNA (for adenine signals); equation for linear fit: $y=221x+2.29$, $R^2=0.917$, $Kb=1.03\times10^4M^{-1}$;

(**c**) FAV/dsDNA (for thymine signals); equation for linear fit: $y=120.45x+241154$, $R^2=0.927$, $Kb=0.20\times10^4M^{-1}$.
